# Reducing Cardiovascular Disease Risk Using Patient Navigators, Denver, Colorado, 2007-2009

**Published:** 2011-10-15

**Authors:** Judith C. Shlay, Beverly Barber, Theresa Mickiewicz, Moises Maravi, Jodi Drisko, Raymond Estacio, Gregory Gutierrez, Christopher Urbina

**Affiliations:** Denver Public Health. Dr Shlay is also affiliated with the Department of Community Health Services, Denver Health and Hospital Authority, Denver, Colorado, and the Department of Family Medicine, University of Colorado, Aurora, Colorado; Denver Public Health, Denver, Colorado; Denver Public Health, Denver, Colorado; Denver Public Health, Denver, Colorado; Drisko Consulting, Kilauea, Hawaii; Denver Health and Hospital Authority, Denver, Colorado, and University of Colorado, Aurora, Colorado; Denver Health and Hospital Authority, Denver, Colorado, and University of Colorado, Aurora, Colorado; Denver Health and Hospital Authority, Denver, Colorado, and University of Colorado, Aurora, Colorado

## Abstract

**Introduction:**

Early identification of cardiovascular disease (CVD) risk is important to reach people in need of treatment. At-risk patients benefit from behavioral counseling in addition to medical therapy. The objective of this study was to determine whether enhanced counseling, using patient navigators trained to counsel patients on CVD risk-reduction strategies and facilitate patient access to community-based lifestyle-change services, reduced CVD risk among at-risk patients in a low-income population.

**Methods:**

We compared clinical characteristics at baseline and 12-month follow-up among 340 intervention and 340 comparison patients from community health centers in Denver, Colorado, between March 2007 and June 2009; all patients had a Framingham risk score (FRS) greater or equal to 10% at baseline. The intervention consisted of patient-centered counseling by bilingual patient navigators. At baseline and at 6-month and 12-month follow-up, we assessed health behaviors of intervention participants. We used an intent-to-treat approach for all analyses and measured significant differences by χ^2^ and *t* tests.

**Results:**

We found significant differences in several clinical outcomes. At follow-up, the mean FRS was lower for the intervention group (mean FRS, 15%) than for the comparison group (mean FRS, 16%); total cholesterol was lower for the intervention group (mean total cholesterol, 183 mg/dL) than for the comparison group (mean total cholesterol, 197 mg/dL). Intervention participants reported significant improvements in some health behaviors at 12-month follow-up, especially nutrition-related behaviors. Behaviors related to tobacco use and cessation attempts did not improve.

**Conclusion:**

Patient navigators may provide some benefit in reducing risk of CVD in a similar population.

## Introduction

The Interheart study demonstrated that 90% of the population-attributable risk of a first myocardial infarction is due to modifiable risk factors (1). Many people in the general population have 1 or more risk factors for cardiovascular disease (CVD), and more than 90% of CVD events occur in people with at least 1 risk factor (1,2).

Early identification of CVD risk is important for treating at-risk people. The National Cholesterol Education Program guidelines recommend using the Framingham risk score (FRS) to identify people with an increased 10-year risk for coronary heart disease (CHD) events (3). The evidence for using the FRS consists of large longitudinal studies in which various models assessing multiple variables estimated the risk of CHD events (4). CHD is a subset of CVD that excludes stroke, peripheral vascular disease, and heart failure.

The appropriate use of a risk score during a provider visit may involve recommendations for preventive lifestyle change in addition to medical therapy (3). However, preventive services are often a low priority in a busy clinical practice because of competing demands, inadequate insurance reimbursement, patient reticence to discuss or follow recommendations, and lack of provider expertise in counseling techniques or knowledge of community-based services (3-7).

Patient navigators have been effective for chronic disease prevention and management activities such as cancer screening and treatment, assessment of primary care services, and cardiovascular health promotion (8-17). The patient-navigator model has been suggested as an approach for facilitating CVD risk-reduction activities in conjunction with other clinical services (18).

The objective of this study was to determine whether enhanced counseling, using patient navigators trained to counsel patients on CVD risk-reduction strategies and facilitate patient access to community-based lifestyle-change services, reduced CVD risk among at-risk patients in a low-income population.

## Methods

### Study design

We used a quasi-experimental pre–post (baseline and 12-month follow-up) design to compare changes in clinical outcomes among intervention participants with changes among a comparison group drawn from the same patient population. We used a nonexperimental pre–post (baseline and 12-month follow-up) design to assess behavioral changes among the intervention participants only. We used an intent-to-treat approach for all analyses.

We enrolled 486 intervention participants from 3 community health centers in the Denver Health and Hospital Authority (DHHA) from March 2007 through June 2009. We collected data on 480 comparison patients from 3 other DHHA community health centers during the same time. This study was reviewed and approved by the Colorado Multiple Institutional Review Board. All intervention participants verbally consented to participate.

### Study setting

DHHA is an urban safety-net health care system that provides services to 25% of Denver residents, including 50% of Denver's children and a large proportion of the city's indigent, vulnerable, and racial/ethnic minority populations. Clinical care components of DHHA include a 500-bed hospital, the 9-1-1 medical response system for the city and county of Denver, 8 federally qualified community health centers, 13 school-based clinics, and Denver Public Health.

### Patient navigators

The patient navigation model has been successful in facilitating care and improving clinical outcomes among cancer patients (12). We revised several components of the model (eg, staff requirements, training) to tailor it to our CVD risk-reduction intervention. Four navigators participated in the intervention. Navigators are bilingual (English and Spanish) peer counselors, certified in core competencies of community health through a 12-week course offered at a local community college. Intervention staff educated the navigators about intervention objectives and purpose, calculation and use of the FRS, and CVD risk-reduction strategies. We trained navigators to identify a participant's readiness to change behaviors, based on the transtheoretical model (19) and to use motivational interviewing techniques to guide the participant's goal-setting process. We provided additional training on human subjects research, privacy practices, smoking cessation, medication adherence, cultural competency, and aggression management. The intervention manager conducted periodic audits on intervention delivery to assess navigator adherence to intervention protocol.

### Intervention

The intervention consisted of a 1-hour counseling session, conducted by a patient navigator at a community health center, plus follow-up telephone calls. During the counseling session, navigators assessed participants' readiness to change behaviors and encouraged them to set goals. The navigator and participant discussed potential CVD risk-reduction activities, focusing on nutrition, physical activity, and smoking cessation. Navigators encouraged participants to continue or increase current activities (eg, walking), provided a free 3-month pass to local Denver County recreation centers, where participants could engage in exercise programs and bilingual nutrition classes, and referred smokers to the Colorado QuitLine, a free smoking-cessation program. Although all intervention activities were independent of any clinical services; the navigator encouraged follow-up with the primary care provider for ongoing clinical care.

The navigators called participants at 1 to 4 weeks and 6 to 10 weeks after enrollment to assist with and encourage the chosen behavior changes or participation in intervention activities; additional calls were made within 6 months of enrollment. On average, each participant received four 15-minute calls.

### Participant eligibility and recruitment

We identified potential intervention participants in a rolling recruitment process through a CVD registry developed for this intervention. We created the registry by collecting the demographic and clinical data needed to calculate an FRS according to methods outlined by Wilson et al (4): age, sex, diabetes diagnosis (based on codes from the *International Classification of Diseases, Ninth Revision, Clinical Modification* [ICD-9-CM] [www.cdc.gov/nchs/icd9.htm] [Appendix]), blood pressure, total cholesterol, high-density lipoprotein (HDL) cholesterol, and smoking status (defined as smoking regularly during the previous 12 months). The FRS estimates 10-year risk for CHD outcomes in people who do not have heart disease (4). We created 3 strata of risk: low risk, <10%; moderate risk, 10% to 20%, and high risk, >20%. We calculated body mass index (BMI) by using weight and height measurements in electronic medical charts. We obtained family history of heart disease by self-report. We collected the data from electronic medical record systems at the 3 intervention sites. Staff updated the registry monthly, allowing identification of potential participants during the 2-year recruitment period. In addition, we extracted the following demographic information from electronic medical records: income and poverty level, race/ethnicity, marital status, level of education, and language spoken.

Eligibility requirements for participation in the intervention included having an FRS 10% or greater, being aged 30 to 64 years, and having an active status at 1 of the 3 intervention community health centers. We defined *active* as having been seen at least twice during the previous 18 months; the most recent visit had to be within the previous 6 months. We excluded patients if they were pregnant or lactating; had a history of coronary artery disease, ischemic cardiomyopathy, myocardial infarction, peripheral vascular disease, symptomatic carotid artery disease, or abdominal aortic aneurysm (based on ICD-9-CM codes [Appendix]); or had a comorbid illness with a life expectancy of less than 12 months.

Intervention staff identified 1,425 potentially eligible participants from the registry during the 2-year study period; 506 people had inaccurate contact information, leaving 919 eligible for inclusion. On a monthly basis, staff sent an introductory letter explaining the intervention to approximately 38 patients. Patient navigators followed up by telephone within 2 weeks to assess interest in participation and invite participants to the 1-hour counseling session. A total of 486 patients verbally consented to participate before counseling began.

Not all intervention participants were included in the final analysis. Three participants who experienced a CHD event after enrollment were not eligible. We defined a CHD event as a diagnosis of angina pectoris, myocardial infarction, coronary insufficiency (ie, unstable angina), or CHD death (based on ICD-9-CM codes [Appendix]); we verified outcomes through chart review. Because the cut-off date for analysis was June 30, 2008, we excluded 143 participants who enrolled after this date; 340 participants were thus included in the final analysis.

### Comparison group eligibility

We used the same eligibility criteria to select a comparison group of 340 patients from 3 nonintervention community health centers. We matched people in the comparison group to intervention participants by age, race/ethnicity, sex, and the month and year in which we identified the participant for inclusion in the registry; we used this group to evaluate the extent to which the changes in the clinical outcomes among intervention participants may have been related to the intervention. We did not contact or counsel people in the comparison group or assess their health behaviors.

### Clinical outcomes

We assessed clinical characteristics at baseline and 12-month follow-up for both intervention and comparison groups. We defined *baseline* as the date of enrollment for the intervention participants and date of record selection for the comparison group. In addition to the data collected to calculate the FRS at baseline and 12-month follow-up, we extracted the following clinical data from electronic medical records for both groups: weight, height, low-density lipoprotein (LDL) cholesterol, and CHD event.

In addition, we collected information on use of medication for lowering blood pressure or cholesterol levels from DHHA pharmacy databases; we defined *baseline pick-up* as pick-up at least twice before study enrollment and *follow-up pick-up* as pick-up at least twice after study enrollment. We calculated medication adherence as a rate: the number of days of medication possession (supply days minus gap days) divided by the number of days of medication exposure (supply days plus gap days).

For certain clinical characteristics, we established dichotomous categories of "at goal" and "not at goal": FRS (at goal, <10%); blood pressure (at goal, <130/80 mm Hg); total cholesterol (at goal, <160 mg/dL); HDL cholesterol (at goal, ≥60 mg/dL); and LDL cholesterol (at goal, <100 mg/dL).

### Behavioral outcomes

We developed a questionnaire to guide motivational interviewing and assess behavioral outcomes after reviewing standardized instruments with demonstrated psychometric properties. The final versions included 30 questions designed to assess 13 measures of nutrition, physical activity (20), stages of change for physical activity (21) and weight loss (22), depression symptoms (23), tobacco use and cessation attempts, and use of nutrition classes and recreation centers. We conducted a pilot test on the questionnaire to determine the time needed to complete it and to assess the target population's comprehension of the questions.

Navigators administered the questionnaire verbally, in English or Spanish, at baseline in person and at 6-month and 12-month follow-up by telephone; completion time averaged 30 minutes. For follow-up, navigators attempted at least 3 telephone calls before considering the participant lost to follow-up. At 12-month follow-up, navigators still attempted to contact participants considered lost to follow-up at 6 months. Among 340 participants eligible for 12-month follow-up, 222 (65%) completed at least 1 follow-up questionnaire at either 6 months or 12 months.

### Statistical analyses

We generated descriptive statistics on baseline demographic and clinical characteristics for the intervention and comparison groups. We performed bivariate analyses and measured significant differences between the groups by using the χ^2^ test for proportional comparisons and the *t* test for continuous variables. For clinical outcomes, we compared changes from baseline to 12 months between the 2 groups. To decrease the possibility of selection bias, we used an intent-to-treat approach for all analyses.

For behavioral outcomes, we assessed changes from baseline to follow-up among the intervention participants only. If the 12-month questionnaire was missing, we used either the baseline questionnaire or the 6-month questionnaire, whichever provided the value closest to the 12-month value. A series of questions determined the number of fruits and vegetables consumed per day and amount of physical activity per week; the goal for each corresponds to current recommendations. A series of questions and predefined algorithms defined the stage of change, which we dichotomized as precontemplation/preparation or action/maintenance. We trichotomized the frequency of consuming high-fat or high-calorie foods. We dichotomized other questions as yes or no. We assessed significant differences between baseline and follow-up in behavioral characteristics by using χ^2^ analyses. We conducted all analyses by using SAS version 9.1.3 software (SAS Institute, Inc, Cary, North Carolina).

## Results

The participation rate for the intervention was 53% (486/919). Mean age was 56 years (SD, 6 y). Most (66%) participants were Hispanic/Latino; 34% spoke Spanish only. Most (76%) had incomes of 150% or less than the federal poverty level. At baseline, almost half of participants smoked, almost half self-reported a family history of heart disease, more than half had hypertension or diabetes, most were overweight or obese (mean BMI, 33 kg/m^2^), and the mean FRS was 15.5%. The comparison group was similar to the intervention group, except for income; only 34% of the comparison group had an income of 150% or less of the poverty level (χ^2^ = 209.5, *P* = .001).

Of the 919 invited to participate, 432 refused participation. Major reasons for refusal included no time (21%), no interest (33%), and failure to keep their appointment with the navigator (33%). Refusers, compared with participants, were significantly more likely to be white non-Hispanic (34% vs 24%), English speakers (79% vs 66%), single (45% vs 35%), and to have a higher baseline total cholesterol level (202 mg/dL vs 192 mg/dL). We found no other demographic or clinical differences.

### Clinical outcomes

At follow-up, the mean FRS was significantly lower for the intervention group than the comparison group; 11.8% of the intervention group was at goal for an FRS, compared with 3.5% of the comparison group (Table 1). At follow-up, the intervention group had lower mean total cholesterol than the comparison group; 29% of the intervention group had total cholesterol less than 160 mg/dL compared with 20% in the comparison group. We found no differences in blood pressure, weight, or HDL cholesterol. At follow-up, mean LDL cholesterol was significantly higher in the intervention group than in the comparison group, but we found no differences between groups in the percentage of those with LDL cholesterol less than 100 mg/dL.

The mean baseline rate of cholesterol medication adherence was 70.1% for the intervention group; it increased by 12.0% to 78.5% at follow-up. The baseline rate for the comparison group was 73.9%; it increased by 2.7% to 75.9% at follow-up. The increases in medication adherence between the 2 groups were significantly different. The mean baseline rate (69.1%) of hypertension medication adherence increased by 9.7% for the intervention group; the mean baseline rate (78.8%) for the comparison group increased significantly by 4.2%.

There were 3 CHD events in the intervention group and 4 CHD events in the comparison group between baseline and follow-up.

### Behavioral outcomes

We found no differences in baseline demographic, behavioral, or clinical characteristics among participants who completed a behavioral questionnaire and those who did not.

We found significant changes in 10 of 13 behavioral measures from baseline to 12 months (Table 2). Participants reported significant improvements in all 6 nutritional measures. While participants did not report improvement in attaining recommended physical activity levels, a significant proportion reported a change from precontemplation/preparation to action/maintenance for weight and exercise. Significantly more participants also reported attending an exercise class at follow-up than at baseline. Behaviors related to tobacco use and cessation attempts did not improve.

## Discussion

This intervention, which used patient navigators to encourage CVD risk-reduction behaviors and connect patients with community-based lifestyle-change services, demonstrated improvements in some clinical and behavioral outcomes for people at risk for CVD. These findings provide evidence for using behavioral approaches to reduce CVD risk in addition to medical therapy.

The most effective approach for CVD prevention is a combination of efforts that works at all levels of influence to create a social and physical environment that is supportive of healthy behaviors. Evidence supports the use of community-based approaches for reducing heart disease risk (24-26). Individually adapted health behavior-change programs improve participation in physical activity, particularly with improved access to locations for physical activity (26). Our intervention linked counseling messages to the participant's stage of readiness to focus on behaviors the patient was most receptive to modify, an effective approach among primary care patients at risk for heart disease (27). Using this strategy, we demonstrated several behavioral changes and improved medication adherence.

Whereas other successful programs (eg, the Vale study), consisting of a more intensive intervention, achieved better reductions in cholesterol levels and other coronary risk factors (28), our intervention, consisting of an average of 5 counseling sessions, resulted in improvement in some clinical and behavioral outcomes. Patient navigation has been used in improving cancer screening and treatment outcomes and increasing the use of health care services among disenfranchised populations (8,9,11,12). Our intervention, which followed the methodology used in cancer-prevention patient-navigation programs (12), was able to enhance the traditional clinical approaches for reducing CVD risk, suggesting that this model could be used for various chronic disease prevention activities (18).

Our study had limitations. For the clinical analyses, lack of randomization increased the possibility of selection bias and confounding. Matching at-risk comparison group members with intervention participants attenuates this possibility; the demographic and clinical characteristics of the 2 groups were similar, except for income levels. The participation rate of 53% may reflect the population often served by safety-net institutions; this population, composed largely of low-income and racial/ethnic minority patients, may harbor distrust in clinical programs. Nevertheless, we observed minimal differences between intervention participants and people who refused to participate. Another possible limitation is that only 65% of the eligible participants completed a follow-up behavioral assessment. We used intent-to-treat analyses to avoid selection bias. We found no differences in baseline characteristics between participants who completed a follow-up assessment and those who did not. Because we did not assess behavior changes in the comparison group, we cannot rule out that behavior changes identified in the intervention group were caused by factors other than the patient navigators. Future studies will need to examine health behaviors of both groups to fully assess the effect of using patient navigators. Finally, because follow-up was only for 1 year, the study was not able to assess the long-term effect of the intervention on CHD events.

Using patient navigators to provide individualized counseling, assistance in goal setting, and linkage to community resources, seems to help intervention participants achieve positive behavior change (particularly related to nutritional activities) and improve several clinical outcomes. This intervention potentially offers a simple way to enhance traditional clinical CVD risk-reduction services. Future studies should consider conducting more rigorous evaluations of the effect of patient navigators on changes in health behaviors.

## Figures and Tables

**Table 1.** Clinical Characteristics Among Intervention and Comparison Groups Eligible for 12-Month Follow-Up (Intent-to-Treat Analysis), Study on Use of Patient Navigators to Reduce Risk for Cardiovascular Disease, Denver, Colorado, 2007-2009

Characteristic	Baseline^a^			12-Month Follow-Up^b^		

	Intervention Group(n = 486)	Comparison Group(n = 480)	*P* Value	Intervention Group(n = 340)	Comparison Group(n = 340)	*P* Value
**Framingham risk score, %^c^**						
Mean (SD)	15.5 (6.2)	15.0 (5.9)	.22^d^	14.8 (6.5)	15.8 (6.0)	.03^d^
<10, No. (%)	0	0	NC	40 (11.8)	12 (3.5)	<.001^e^
10-20, No. (%)	408 (84)	420 (87)	.11^e^	255 (75.0)	277 (81.5)	.04^e^
>20, No. (%)	78 (16)	60 (13)	.11^e^	45 (13.2)	51(15.0)	.50^e^
**Blood pressure, mm Hg**						
Mean (SD)	138/81 (18/11)	140/84 (20/10)	.13/<.001^d^	139/81(18/12)	139/83 (19/9)	.9/.02^d^
<130/80, No. (%)	119 (24.5)	93 (19.4)	.06^e^	80 (23.5)	66 (19.4)	.19^e^
≥130/80, No. (%)	367 (75.5)	387 (80.6)		260 (76.5)	274 (80.6)	
**Weight, lb**						
Mean (SD)	194 (43)	191 (46)	.22^d^	195 (44)	191 (7)	.28^d^
**Total cholesterol, mg/dL**						
Mean (SD)	192 (40)	197 (48)	.07^d^	183 (44)	197 (49)	<.001^d^
<160, No. (%)	76 (16)	86 (18)	.34^e^	99 (29)	68 (20)	<.001^e^
≥160, No. (%)	410 (84)	394 (82)		241 (71)	272 (80)	
**High-density lipoprotein cholesterol, mg/dL**						
Mean (SD)	44 (10)	44 (12)	.96^d^	44 (10)	44 (12)	.74^d^
<60, No. (%)	457 (94)	443 (92)	.28^e^	316 (93)	312 (92)	.56^e^
≥60, No. (%)	29 (6)	37 (8)		24 (7)	28 (8)	
**Low-density lipoprotein cholesterol, mg/dL**						
Mean (SD)	114 (37)	112 (39)	.39^d^	118 (37)	111 (40)	.02^d^
<100, No. (%)	181 (38)	185 (40)	.63^e^	120 (36)	133 (40)	.21^e^
≥100, No. (%)	294 (62)	282 (60)		218 (64)	198 (60)	

Abbreviation: NC, not calculated; SD, standard deviation.

a Intervention group includes all people enrolled in the intervention; intervention participants received services from a patient navigator, including counseling and referral to community-based lifestyle behavior-change services. Baseline comparison group includes people who met eligibility requirements for enrollment but did not receive the intervention.

b Includes intervention participants and comparison group members eligible for 12-month follow-up. Not eligible for follow-up were 3 intervention participants and 4 comparison group members who had a coronary heart disease event. If no clinical follow-up was completed, we assumed no change occurred and used baseline values.

c The Framingham risk score estimates 10-year risk for coronary heart disease outcomes in people who do not have heart disease (4).

d *P* value calculated by using pooled *t* test with equal variances; it compares intervention and comparison groups.

e *P* value calculated by using χ^2^ test; it compares intervention and comparison groups.

**Table 2.** Change in Behavioral Characteristics Among Intervention Participants Eligible for 12-Month Follow-Up (Intent-to-Treat Analysis), Study on Use of Patient Navigators to Reduce Risk for Cardiovascular Disease, Denver, Colorado, 2007-2009

**Characteristic**	Baseline,^a^ No. (%) (n = 343)	12-Month Follow-Up,^b^ No. (%) (n = 340)	*P* Value^c^
**Consume ≥5 servings of fruits and/or vegetables per day**			
Yes	7 (2.0)	32 (9.8)	<.001
No	336 (98.0)	294 (90.3)	
**During the past 7 days, number of times drank soda**			
None	167 (48.8)	206 (63.4)	<.001
≤Once per day	135 (39.5)	96 (29.5)	
≥Twice per day	40 (11.7)	23 (7.1)	
**During the past 7 days, number of times pastry eaten**			
None	84 (24.5)	147 (45.1)	<.001
≤Once per day	237 (69.1)	166 (50.9)	
≥Twice per day	22 (6.4)	13 (4.0)	
**During the past 7 days, number of times fried foods eaten**			
None	83 (24.3)	133 (40.1)	<.001
≤Once per day	232 (67.8)	183 (56.1)	
≥Twice per day	27 (7.9)	10 (3.1)	
**During the past 7 days, how often did you read labels?**			
Never or rarely	212 (61.8)	152 (46.8)	<.001
Sometimes or more often	131 (38.2)	173 (53.2)	
**Attended a nutrition class in the last 6 months**			
Yes	13 (3.8)	49 (15.0)	<.001
No	330 (96.2)	277 (85.0)	
**Stage of change regarding weight**			
Precontemplation/preparation	120 (35.5)	78 (24.5)	.002
Action/maintenance	218 (64.5)	240 (75.5)	
**Physical activity level^d^**			
At or above recommended level	135 (40.5)	129 (38.2)	.64
Below recommended level	198 (59.5)	209 (61.8)	
**Stage of change regarding exercise**			
Precontemplation/preparation	82 (32.0)	47 (19.0)	.001
Action/maintenance	174 (68.0)	201 (81.0)	
**Attended an exercise class in the last 6 months**			
Yes	35 (10.2)	94 (29.1)	<.001
No	307 (89.8)	229 (70.9)	
**Current smoker**			
Yes	117 (34.2)	97 (30.8)	.32
No	225 (65.8)	218 (69.2)	
**Among smokers, tried to quit in last 12 months**			
Yes	62 (54.9)	47 (49.5)	.46
No	51 (45.1)	48 (50.5)	
**Over the last 2 weeks, felt depressed for several days or more**			
Yes	188 (55.0)	109 (33.5)	<.001
No	154 (45.0)	216 (66.5)	

a At baseline, intervention group includes all people enrolled in the intervention; intervention participants received services from a patient navigator, including counseling and referral to community-based lifestyle behavior-change services. Baseline comparison group includes people who met eligibility requirements for enrollment but did not receive the intervention.

b Includes all intervention participants eligible for the 12-month follow-up interview but excludes 3 who had a coronary heart disease event. If the 12-month questionnaire was missing, we used either the baseline questionnaire or the 6-month questionnaire, whichever provided the value closest to the 12-month value.

c *P* value calculated by using χ^2^ test; it compares baseline with 12-month follow-up.

d Recommended physical activity level defined as 30 minutes of moderate activity or walking 5 or more days per week.

## Appendix. Diagnosis Codes Used to Assess Eligibility for Study on Patient Navigators, Denver, Colorado, 2007-2009

Codes were obtained from the *International Classification of Diseases, Ninth Revision, Clinical Modification* (ICD-9-CM) (www.cdc.gov/nchs/icd9.htm).

**Table:**

**Diagnosis**	**Code**
**Cardiomyopathy**	414.8
**Coronary artery disease**	411.0, 411.1, 411.8, 411.81, 411.89, 414.0, 414.00, 414.01, 414.02, 414.03, 414.04, 414.05, 414.06, 414.07, 414.10, 414.11, 414.12, 414.8, 414.9
**Myocardial infarction**	410.0, 410.00, 410.01, 410.02, 410.1, 410.10, 410.11, 410.12, 410.2, 410.20, 410.21, 410.22, 410.3, 410.30, 410.31, 410.32, 410.4, 410.40, 410.41, 410.42, 410.5, 410.50, 410.51, 410.52, 410.6, 410.60, 410.61, 410.62, 410.7, 410.70, 410.71, 410.72, 410.8, 410.80, 410.81, 410.82, 410.9, 410.90, 410.91, 410.92
**Peripheral vascular disease**	440.0, 440.1, 440.2, 440.20, 440.21, 440.22, 440.23, 440.24, 440.29, 440.30, 440.31, 440.32, 440.8, 440.9, 443.21, 443.89, 443.9
**Symptomatic carotid artery disease**	433.1, 433.11, 437.3, 442.81, 900.00, 900.01, 900.02, 900.03, 996.1
**Abdominal aortic aneurysm**	093.0, 441.3, 441.5, 441.9
**Diabetes**	250, 250.00, 250.01, 250.02, 250.03, 250.10, 250.11, 250.12, 250.13, 250.20, 250.21, 250.22, 250.23, 250.30, 250.31, 250.32, 250.33, 250.40, 250.41, 250.42, 250.43, 250.50, 250.51, 250.52, 250.53, 250.60, 250.61, 250.62, 250.63, 250.70, 250.71, 250.72, 250.73, 250.80, 250.81, 250.82, 250.83, 250.90, 250.91, 250.92, 250.93, 648.00, 648.01, 648.02, 648.03, 648.04
